# What effect do substorms have on the content of the radiation belts?

**DOI:** 10.1002/2016JA022620

**Published:** 2016-07-09

**Authors:** C. Forsyth, I. J. Rae, K. R. Murphy, M. P. Freeman, C.‐L. Huang, H. E. Spence, A. J. Boyd, J. C. Coxon, C. M. Jackman, N. M. E. Kalmoni, C. E. J. Watt

**Affiliations:** ^1^ Mullard Space Science LaboratoryUniversity College LondonDorkingUK; ^2^ NASA Goddard Space Flight CenterGreenbeltMarylandUSA; ^3^ British Antarctic SurveyCambridgeUK; ^4^ Space Science CenterUniversity of New HampshireDurhamNew HampshireUSA; ^5^ New Mexico ConsortiumLos AlamosNew MexicoUSA; ^6^ School of Physics and AstronomyUniversity of SouthamptonSouthamptonUK; ^7^ Department of MeteorologyUniversity of ReadingReadingUK

**Keywords:** substorm, radiation belts, enhancements, losses

## Abstract

Substorms are fundamental and dynamic processes in the magnetosphere, converting captured solar wind magnetic energy into plasma energy. These substorms have been suggested to be a key driver of energetic electron enhancements in the outer radiation belts. Substorms inject a keV “seed” population into the inner magnetosphere which is subsequently energized through wave‐particle interactions up to relativistic energies; however, the extent to which substorms enhance the radiation belts, either directly or indirectly, has never before been quantified. In this study, we examine increases and decreases in the total radiation belt electron content (TRBEC) following substorms and geomagnetically quiet intervals. Our results show that the radiation belts are inherently lossy, shown by a negative median change in TRBEC at all intervals following substorms and quiet intervals. However, there are up to 3 times as many increases in TRBEC following substorm intervals. There is a lag of 1–3 days between the substorm or quiet intervals and their greatest effect on radiation belt content, shown in the difference between the occurrence of increases and losses in TRBEC following substorms and quiet intervals, the mean change in TRBEC following substorms or quiet intervals, and the cross correlation between SuperMAG *AL* (SML) and TRBEC. However, there is a statistically significant effect on the occurrence of increases and decreases in TRBEC up to a lag of 6 days. Increases in radiation belt content show a significant correlation with SML and *SYM‐H*, but decreases in the radiation belt show no apparent link with magnetospheric activity levels.

## Key Points

- The radiation belts are inherently lossy
- Substorms increase the likelihood of an increase in the radiation belts to 50%
- The radiation belts have a memory of magnetospheric activity for up to 6 days

## 1 Introduction

Earth's radiation belts consist of trapped electrons and protons at MeV energies drift bouncing around the Earth at radial distances between 1000 km and 6 *R_E_*. The flux of particles in the radiation belts is the result of competing enhancement and loss mechanisms and can vary by orders of magnitude. Enhancements in the radiation belt population occur through direct injection of energized particles, radial diffusion, and energization through conservation of adiabatic invariants, or wave‐particle interactions, whereas losses from the radiation belts generally occur via pitch angle scattering, adiabatic diffusion, or particle drift paths intersecting the magnetopause (see reviews by *Millan and Thorne* [2007], *Ebihara and Miyoshi* [2011], and *Ukhorskiy and Sitnov* [2012]). While the loss and acceleration mechanisms have been long studied, the phenomenological processes which lead to radiation belt increases and losses remain unclear [e.g., *Reeves et al*., 2003] and thus are a key target for the Van Allen Probes mission [*Mauk et al*., 2012].

One mechanism that is thought to be a major contributor to increases in the radiation belt is substorm particle injections. Rather than directly injecting MeV energy particles into the radiation belts, substorms are thought to provide a low‐energy population of keV electrons which are subsequently accelerated to higher energies [e.g., *Baker et al*., 1998; *Horne and Thorne*, 1998; *Fok et al*., 2001; *Meredith et al*., 2002, 2003a, 2003b, 2003c].

It has long been established that particle injections into the inner magnetosphere and outer radiation belts occur during substorms [e.g., *Pfitzer and Winckler*, 1969; *Akasofu*, 1968; *Reeves et al*., 1990; *Borovsky et al*., 1993; *Baker et al*., 1998; *Fok et al*., 2001]. Early studies suggested that these injections occurred across a wide injection boundary [e.g., *Mauk and McIlwain*, 1974; *Konradi et al*., 1975; *Mauk and Meng*, 1983; *Moore et al*., 1981]; however, later studies have shown that the injection region is limited in magnetic local time extent [*Reeves et al*., 1990], with a greater proportion of injections occurring in the premidnight sector [*Birn et al*., 1997a, 1997b; *Thomsen et al*., 2001; *Gabrielse et al*., 2014]. The injected particles do not, on average, have sufficient energy to significantly enhance the relativistic particle population. Although the injection of MeV particles directly into the outer radiation belts by substorm dipolarizations has been reported [e.g., *Dai et al*., 2014], *Baker et al*. [1979] found that more than 80% of substorms did not result in an injection of >0.3 MeV protons at geosynchronous orbit. Instead, the substorm injection is considered to provide a “seed” population of keV particles [*Baker et al*., 1998] to the outer radiation belt. This seed population is anisotropic and unstable to the generation of whistler mode chorus waves [*Li et al*., 2010, and references therein]. The seed population is subsequently locally accelerated through wave‐particle interactions with these whistler mode chorus waves [*Horne and Thorne*, 1998; *Summers et al*., 1998; *Horne et al*., 2005a, 2005b; *Li et al*., 2007; *Jaynes et al*., 2015] up to relativistic energies. Hence, substorms are thought to be the source of the electron seed population and the source of the wave growth that provides the acceleration of these particles to relativistic energies.

*Reeves et al*. [2003] have shown that the radiation belts do not show a consistent response to storm activity, with the outer belt relativistic electron fluxes increasing, decreasing or remaining invariant for storms with a similar *Dst* profile. In this paper we ask whether a similar result can be found for substorms. To that end, we assess the extent to which substorms enhance the content of the electron radiation belts by comparing times of increases and decreases in the radiation belts with substorm activity. We also examine how the increase and decrease of the radiation belt content compare with a measure of the size of the substorm. Our observations show that 50% of substorm intervals are followed by an increase in radiation belt content and 50% by a decrease. To fully understand variations in the radiation belt fluxes, any phenomenological framework or physical model must explain both the enhancements and reductions of the radiation belts following substorms.

## 2 Instrumentation and Methodology

The Van Allen Probes [*Mauk et al*., 2012] are a pair of identical spacecraft in 500 × 30,600 km near‐equatorial orbits of the Earth. The orbits of the two spacecraft are slightly different, such that the separation between the spacecraft changes with time. Each spacecraft has an identical suite of five instruments designed to measure the radiation belt plasma and electromagnetic fields.

We use data from the Van Allen Probes Magnetic Electron Ion Spectrometer (MagEIS) [*Blake et al*., 2013] instrument in the Energetic Particle, Composition, and Thermal Plasma suite (ECT) [*Spence et al*., 2013] from 1 January to 31 December 2013. From these data, and building on the ideas of *Baker et al*. [2004], the total radiation belt electron content (TRBEC) has been calculated. The calculation is detailed in *Boyd* [2016] and Huang et al (Spatial, temporal and energy dependence of total radiation belt electron content, GRL, manuscript in preparation, 2016). In summary, TRBEC is calculated using the Jacobian determinant calculated using the three action integrals of the electrons' three quasiperiodic motions, *J*1, *J*2, and *J*3, with respect to gyration, bounce motion, and drift motion [*Schulz*, 1991]. From this, the number of electrons can be calculated as

$$N=∭{\left(2\pi \right)}^{3}f\left(\mu ,K,L^{*}\right)\frac{8\sqrt{2}\pi^{2}m_{0}^{3/2}\mu_{0}}{R_{E}}\frac{\sqrt{\mu }}{L^{*2}}d\mu dKdL^{*}$$

By integrating across an appropriate range of the first adiabatic invariant (*μ*), pitch angle (*K*), and *L* ^*^ along each half orbit of the spacecraft, the TRBEC for different energies of electrons can be calculated. In this case, the distributions are integrated between *μ* = 1000–2000 MeV/G to give an estimate of the number of particles in the “core” radiation belt population. This corresponds to particles in the energy range 0.3–6 MeV at *L* = 3–6. This provides an estimate of the radiation belt content approximately every 3 h. These data are then interpolated onto a regular 3 h timeline.

In order to determine changes in TRBEC as a function of substorm occurrence, we require a reliable estimate of substorm expansion and recovery phase times that is continuous over the period covered by the Van Allen Probes TRBEC data. We define substorm intervals using the SOPHIE technique [*Forsyth et al*., 2015]. This technique provides the onset times of expansion and recovery phases, as well as substorm intensifications (expansion phases directly following recovery phases), based on the SuperMAG *AL* (SML) index [*Newell and Gjerloev*, 2011]. In summary, the technique identifies the following: (1) expansion phase as a negative rate of change in smoothed SML below a determined threshold, (2) recovery phase as a positive rate of change in smoothed SML above a different determined threshold, and (3) possible growth phase at any other time.

The SOPHIE technique also determines those intervals in which SML shows substorm‐like characteristics, but in which the SML variations are mirrored by the SuperMAG *AU* (SMU) index. These are interpreted as being intervals of enhanced magnetospheric convection and not substorm events. The thresholds for the expansion and recovery phase identifications are calculated from a specified percentile of the rates of change in SML. *Forsyth et al*. [2015] found that the expansion phase onsets determined using the 75th percentile gave good agreement with existing auroral‐based [*Frey and Mende*, 2006; *Liou et al*., 2001] and magnetometer‐based [*Newell and Gjerloev*, 2011] substorm onset lists. Thus, we use this percentile for all phase identifications in this study.

In order to compare substorm activity with TRBEC and changes in TRBEC, we identify whether one or more expansion or recovery phase onsets occurred within the 3 h window of the TRBEC data. In the following, these intervals are described as “substorm intervals.” If there was no expansion or recovery phase onset and no evidence of an enhanced convection event within the time window, the interval is considered to be a “quiet interval.”

We also compare TRBEC and changes in TRBEC with SML and *SYM‐H*. SML provides a measure of substorm activity and the strength. Similarly, *SYM‐H* provides a measure of storm activity and strength. In order to compare these data with the TRBEC data, we downsample SML and *SYM‐H* by calculating the mean of the data over the 3 h corresponding to each TRBEC data point.

### 2.1 Comparison Between Radiation Belt Content and Substorms

**Figure 1:**
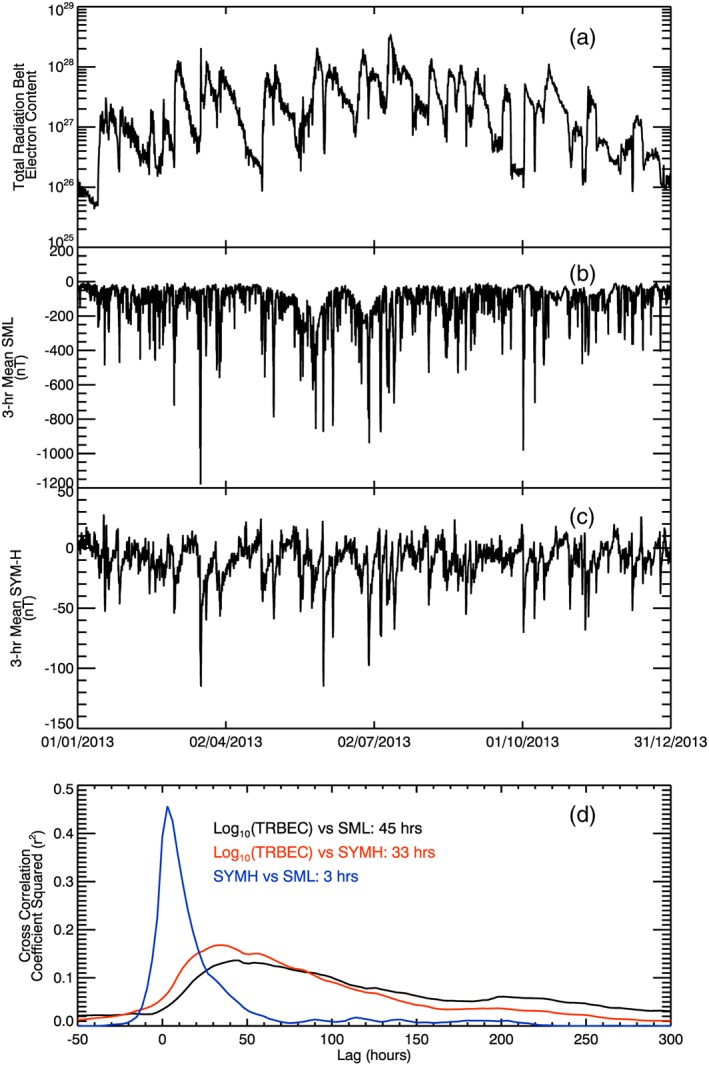
Plot showing (a) the total radiation belt electron content of 1000–2000 MeV/G electrons as measured by the Van Allen Probes and (b) 3 h averaged (mean) SuperMAG *AL* (SML) index and (c) *SYM‐H* against universal time for 2013. (d) The Pearson's correlation coefficient determined for different lags between TRBEC and SML (black) , TRBEC and *SYM‐H* (red), and *SYM‐H* and SML (blue). The lag corresponding to the peak correlation coefficient is shown in the panel. The data show that TRBEC is weakly correlated with SML and *SYM‐H* at 45 and 33 h, respectively (TRBEC varying after SML or *SYM‐H*), and that *SYM‐H* and SML are moderately correlated at a lag of 3 h.

Figure 1 provides an overview of the data used in this study and shows (a) the total radiation belt electron content, (b) the 3 h mean of the SML index, and (c) the 3 h mean of *SYM‐H* between 1 January and 31 December 2013 inclusive. It is these data that we will use throughout this paper. Figure 1d shows the cross correlation of these data using the square of the Pearson's correlation coefficient (*r* ^2^) plotted against lag times. The correlation between TRBEC and SML is shown in black, the correlation between TRBEC and *SYM‐H* is in red, and the correlation between *SYM‐H* and SML is in blue. Positive lags indicate that changes in TRBEC follow changes in SML or *SYM‐H* and that changes in *SYM‐H* follow changes in SML. The time of the maximum cross correlation is given in the figure.

Figure 1a shows that during 2013 TRBEC varied by over 2 orders of magnitude and on a variety of times cales, although the most noticeable on this scale are the large‐scale variations that occur approximately twice per month. Figure 1b shows that SML tends to vary on shorter time scales, as we would expect for substorms that occur, on average, four times per day, and Figure 1c shows that *SYM‐H* varies on a slightly longer period, as expected for storm activity. Figure 1d shows that there is a weak (<15%) anticorrelation between TRBEC and both SML and *SYM‐H*, with the cross correlations maximizing for TRBEC lagging SML by 45 h and lagging *SYM‐H* by 33 h. This is consistent with the framework in which particles injected during a substorm take some time to be energized to relativistic energies, but the time scales are longer than the 24 h time scale predicted by *Horne et al*. [2005a]. Figure 1d also shows that the 3 h mean *SYM‐H* and SML are 45% correlated with *SYM‐H* lagging SML by 3 h. It is therefore difficult to deconvolve substorm effects from storm effects on a 3 h time scale. In the analysis presented later in this paper, we account for this by comparing changes in TRBEC with SML and *SYM‐H* in parallel.

In order to determine the extent to which substorms influence the electron content of the radiation belts, we determine the change in TRBEC following a substorm or quiet interval. The change in TRBEC is calculated as ΔTRBEC = TRBEC(*t*_0_ + *T*) − TRBEC(*t*_0_), where *T* is the differencing time lag and *t*_0_ is the reference time (the time of the substorm or quiet window from which we wish to know the change in TRBEC). This change is thus the net or time‐integrated change in the radiation belt content from the reference event. The data at each time step form a 2 × 2 contingency table. An example contingency table is shown in Table 1, showing the number of increases (column 1) and decreases (column 2) in TRBEC 24 h following substorms (row 1) or quiet intervals (row 2). By dividing each row element by that row's total, we are able to determine the proportion of increases or decreases following substorm or quiet intervals. In this case, approximately 50% of substorms are followed by an increase in TRBEC, whereas only 30% of quiet intervals are followed by an increase in TRBEC. Alternatively, we can rearrange the data to examine the proportion of increases or decreases preceded by a substorm or quiet interval, as shown in Table 2. This shows, from the same data, that ~75% of increases in TRBEC are preceded by a substorm interval 24 h beforehand, whereas only 50% of decreases are preceded by a substorm. In order to determine whether the ratios of increases to decrease of TRBEC following substorms or quiet intervals are statistically significantly different, we need to compare them to a null hypothesis that substorms have no effect on increases or decreases in the radiation belts. The expected values from this hypothesis for each cell in the table are the product of the row total and column total divided by the total number of observations. We can also thus use the *χ* ^2^ statistic to assess whether the observed occurrences are statistically significantly different from expected values assuming the null hypothesis.

**Table 1:** Contingency Table of TRBEC Increases and Decreases 24 h Following Substorm or Quiet Intervals

	Observed Values		Proportion of Substorm/Quiet Observations		Expected Values for Null Hypothesis (No Relation Between Increases or Decreases With Substorms or Quiet Intervals)	
	TRBEC Increase	TRBEC Decrease	TRBEC Increase	TRBEC Decrease	TRBEC Increase	TRBEC Decrease
Substorm interval	851	838	0.504	0.496	714	975
Quiet interval	303	738	0.291	0.709	440	601

**Table 2:** Contingency Table of TRBEC Increases and Decreases 24 h Following Substorm or Quiet Intervals

	Observed Values		Proportion of Increases or Decreases Following:		Expected Values for Null Hypothesis (No Relation Between Increases or Decreases With Substorms or Quiet Intervals)	
	Substorm Interval	Quiet Interval	Substorm Interval	Quiet Interval	Substorm Interval	Quiet Interval
TRBEC increase	851	303	0.737	0.263	714	440
TRBEC decrease	838	738	0.532	0.468	975	601

**Figure 2:**
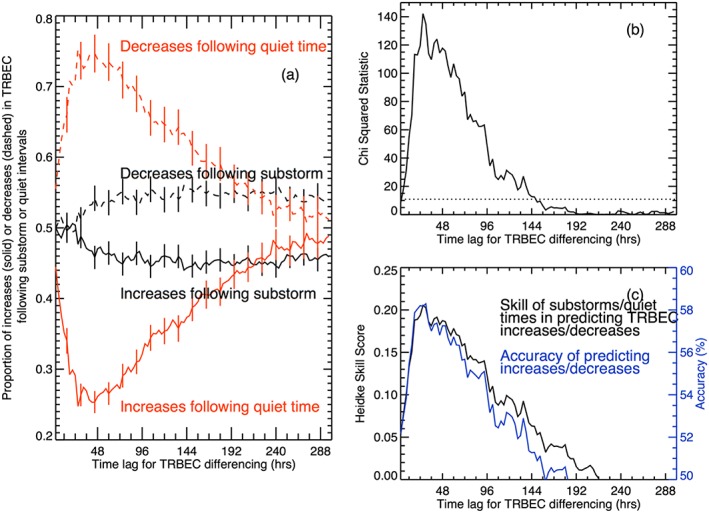
(a) The proportion of quiet intervals (red) and substorm intervals (black) that are followed by an increase (solid lines) or decrease (dashed lines) in TRBEC for different lags. The change in TRBEC is calculated as TRBEC(*t*_0_ + d*t*) − TRBEC(*t*_0_) where d*t* is the lag. (b) Plot of the *χ* ^2^ statistic showing the statistical significance of the differences in the proportions in Figure 2a. The dotted horizontal line shows the lower limit for the results to be significant beyond the 99.9% level. (c) The Heidke Skill Score (black) and accuracy (blue) obtained for using substorms or quiet times to predict increases or decreases in TRBEC. The data show that up to 33 h after a substorm interval, 50% of events show an increase in TRBEC and 50% show a decrease, whereas following a quiet interval, there is up to a 75% chance of TRBEC decreasing.

Figure 2a shows the proportion of substorm intervals (black) and quiet intervals (red) that were followed by an increase (solid line) or decrease (dashed line) in TRBEC, for different time lags (*T*), following the presentation of data in Table 1. It shows that for each 3 h interval up to 33 h following substorm intervals there was an ~50% chance that TRBEC was increased above the level during the substorm interval, after which time the likelihood of a decrease in TRBEC increased, tending toward 55% after 144 h. In contrast, the likelihood of a decrease in TRBEC following a quiet interval was 55% in the 3 h following the quiet interval but steadily increased to ~75% at 45 h following the quiet interval. Using the *Z* statistic (*Z* = (*a* − *b*)/(*a* + *b*)^0.5^, not shown), we find that there were statistically significantly more decreases than increases (at the 99.9% level) following quiet intervals up to a lag of 219 h. Following substorms, the difference between increases and decreases in TRBEC was statistically insignificant up to a lag of 69 h, in keeping with the 50/50 split shown, after which time there were significantly more decreases. In summary, our results show that there are statistically significantly no more increases than decreases following substorms up to 69 h after the reference interval but statistically significantly more decreases following quiet intervals up to 219 h (9 days) after the reference interval.

Using the contingency table analysis, we calculate the *χ* ^2^ statistic at each time lag (Figure 2b). The *χ* ^2^ statistic was much greater than the 99.9% significance level for lags between 6 h and 144 h and had a broad peak (*χ* ^2^ > 100) between 0.5 and 2.5 days. Over a lag of ~0–1 day, the increase in *χ* ^2^ is driven by an increasing proportion of decreases following quiet intervals while the proportion of increases following substorms was constant. Over a lag of 1–2 days, the proportion of decreases following quiet intervals is approximately constant, while the proportion of increases following substorms decreases, resulting in a decrease in *χ* ^2^. Finally, between over a lag of 2–6 days, the change in *χ* ^2^ is driven by a decrease in the proportion of decreases following quiet intervals.

We are also able to test whether substorms are a good predictor of increases (and conversely that quiet intervals are good predictors of decreases) by calculating the Heidke Skill Score (HSS) [*Heidke*, 1926] and the accuracy of the prediction. For the interested reader, these are described in the supporting information. Under the premise that substorms lead to increases in TRBEC and that quiet intervals lead to decreases in TRBEC, we can take the occurrence of substorm and quiet intervals to be a forecast of increases or decreases in TRBEC and assess the skill of this forecast in terms of the HSS and accuracy as a function of time lag. In this analysis, we are only assessing the occurrence of increases or decreases, not the size of the change in TRBEC. The HSS (black) and accuracy (red) of using substorms or quiet times to predict increase or decreases in TRBEC is shown in Figure 2c. For HSS > 0, substorm intervals have some skill in predicting increases in TRBEC (the maximum possible HSS is 1). Figure 2c shows that the calculated HSS is greater than 0 for all time lags up to 216 h intervals and peaks at 0.21 after 27 h, in keeping with the maximum in the *χ* ^2^ statistic. Thus, using substorm and quiet intervals to predict radiation belt increases and decreases has some skill over that interval. The accuracy of the prediction peaks at 58% for a time lag of 30 h but remains above 50% up to a lag of 183 h.

Overall, the analysis shown in Figure 2 indicates that there is a statistically significantly higher likelihood of an increase in the radiation belts up to 6 days following a substorm than in the same period following a quiet interval. The difference in the occurrence of increases or decreases in TRBEC following substorms or quiet intervals is most significant 0.5–2.5 days following the substorm or quiet interval, in keeping with the 48 h cross correlation lag between SML and TRBEC. Up to half of the substorm intervals were followed by an increase in TRBEC, and up three quarters of quiet intervals were followed by a decrease in TRBEC. Similarly, up to three quarters of increases in TRBEC were preceded by a substorm, as were half the decreases in TRBEC.

The results of Figure 2 show that there is a statistically significant difference in the response of the radiation belts to substorms and quiet intervals but does not show which has the greater influence. If we take assume that the magnetosphere is normally quiet times and that substorms are a perturbation to this quiet system, then the results of Figure 2 can be interpreted as showing that substorm activity doubles the likelihood of the radiation belts increasing. However, the “memory” in the radiation belts of substorms and quiet times is far longer than the initial 3 h interval. For up to 6 days after substorms and quiet times, the proportion of increases and decreases in TRBEC is statistically significantly different from those expected if substorms and quiet intervals have no effect on the radiation belts.

**Figure 3:**
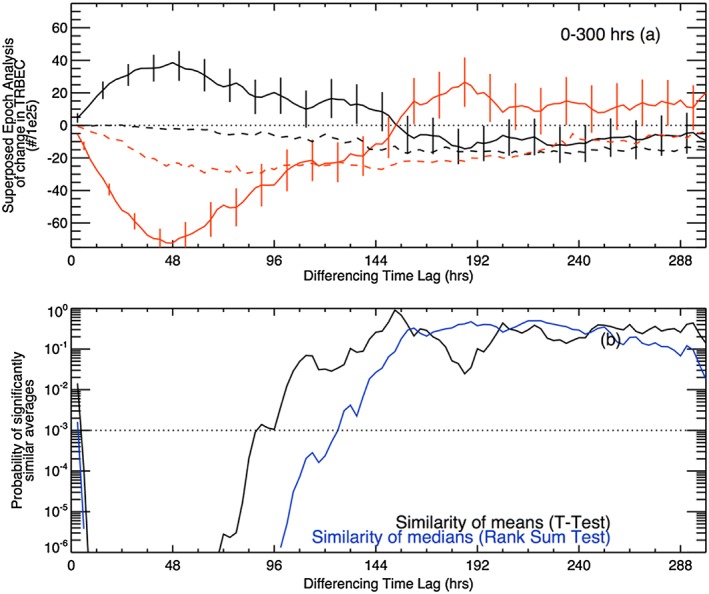
(a) Superposed epoch analysis of the change in TRBEC following substorm (black) and quiet (red) intervals. (b) The probability that the means (black) and medians (blue) of the change in TRBEC following substorms and quiet intervals are statistically similar. Points below the horizontal line have a probability of less than 0.1% or being similar or are statistically significantly different at the 99.9% level. This value is calculated from the Student's *T* test for the means and Wilcoxon‐Mann‐Whitney Rank Sum Test for the medians.

**Figure 4:**
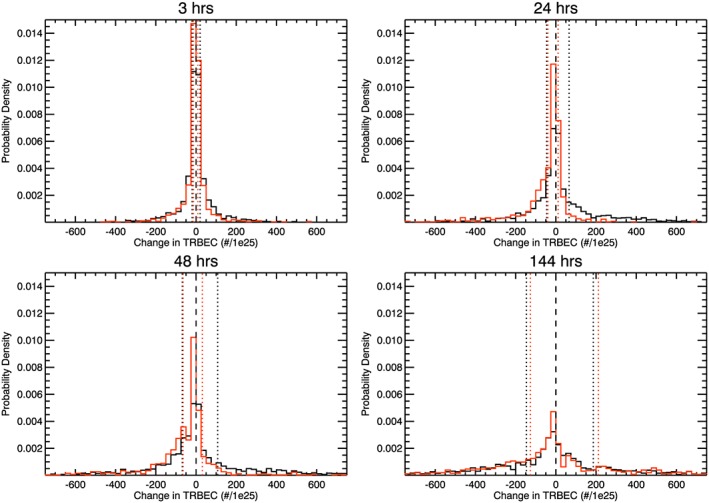
Histograms of the probability density of changes in following substorms (black) and quiet intervals (red) at lags of (a) 3 h, (b) 24 h, (c) 48 h, and (d) 144 h. The vertical dotted lines show the median increase and decrease in TRBEC. The plots show that the distribution of decreases in TRBEC is largely similar following substorms and quiet intervals at all lags, whereas substorms enhance the distribution of increases in TRBEC. However, this enhancement is sensitive to the time from the substorm interval and has all but vanished by a lag of 144 h.

Figure 3a shows a superposed epoch analysis of the change of TRBEC following substorm (black) and quiet (red) intervals. The solid lines show the results of the analysis taking the means of the changes in TRBEC at each time lag, and the dashed lines show the medians. The error bars on the solid lines show the standard error in the means. Figure 3b shows the probability that the mean (black) or median (blue) change in TRBEC following substorms or quiet intervals is statistically significantly similar. These are the *P* values resulting from Student's *T* test of the difference in the means and the Wilcoxon‐Mann‐Whitney Rank Sum Test of the difference in the medians. Figure 4 shows the probability density of a change in TRBEC following substorm intervals (black) and quiet intervals (red) at lags of (a) 3 h, (b) 24 h, (c) 48 h, and (d) 144 h.

Figures 3 and 4 show that the radiation belts are inherently lossy: the median changes in TRBEC are negative at all times following both substorm and quiet intervals (Figure 3), which is replicated in the highest probability densities occurring for negative changes in TRBEC (Figure 4). However, Figure 3a shows that the mean change in TRBEC following substorm periods is positive up to a lag of 144 h. As such, the distribution of increases in TRBEC following substorms has a much longer tail than the distribution of decreases. The effect of this can be seen as the sawtooth‐like profile of TRBEC in Figure 1a; the increases occurred over short periods, whereas the decreases extend over several days.

Following substorm intervals, the mean change in TRBEC is positive, peaking 48 h after the substorm interval. Similarly, the mean change in TRBEC following quiet intervals is negative, minimizing 48 h after the quiet interval, although we note that these peak and trough of the mean changes are broad, as per the results of Figure 2. This is consistent (to within one data point) with the cross correlation between SML and TRBEC shown in Figure 1. In contrast, while the median change in TRBEC following quiet intervals also shows a decrease, the median change in TRBEC following substorms shows little or no increase. The significance tests shown in Figure 3b show that the difference in the mean changes in TRBEC following substorms or quiet interval is statistically significant beyond the 99.9% level up to a lag of 90 h, whereas the median changes are statistically significantly different up to a lag of 129 h.

Comparing the distributions of changes in TRBEC, we see that the distributions of positive changes in TRBEC are elevated following substorms, with respect to the following quiet intervals, at lags of 3, 24, and 48. After 144 h, the distributions of changes in TRBEC following substorms and quiet intervals are more similar, in keeping with the lack of statistically significant differences in the averages and occurrences shown above. The probability distributions shown indicate that the losses from the radiation belt are slightly elevated following quiet intervals as compared to following substorm intervals, but not to the extent that the increases differ following substorms compared to quiet times.

The vertical dotted lines in Figure 4 show the median increases and decreases of TRBEC following substorms (black) and quiet intervals (red). Dividing each change in TRBEC by the time lag gives a mean rate of change, from which we convert the dotted lines to the median mean rates of change (shown in Table 3). There is no statistically significant difference in median mean rate of decrease in TRBEC following substorms and quiet intervals at lags of 3, 24, and 48 h. In contrast, the median mean rate of increase in TRBEC is statistically significantly greater following substorms than following quiet times. It should be noted that the analysis we have presented effectively low‐pass filters the variations in TRBEC with increasing window length. As such, directly comparing the average rates of the changes is somewhat problematic. For a window length of 3 h, the rates of change can be dominated by the uncertainty in the data, whereas larger windows may smooth out significant but short‐term variations.

**Table 3:** Median Mean Rates of Change of TRBEC for Different Lags^a^

	3 h	24 h	48 h	144 h
Rate of increase following substorms	159	66	54	31
Rate of increase following quiet intervals	73	11	15	35
Rate of decrease following substorms	−156	−46	−34	−24
Rate of decrease following quiet intervals	−101	−40	−32	−21

a Results are given in units of 1E25/day.

**Figure 5:**
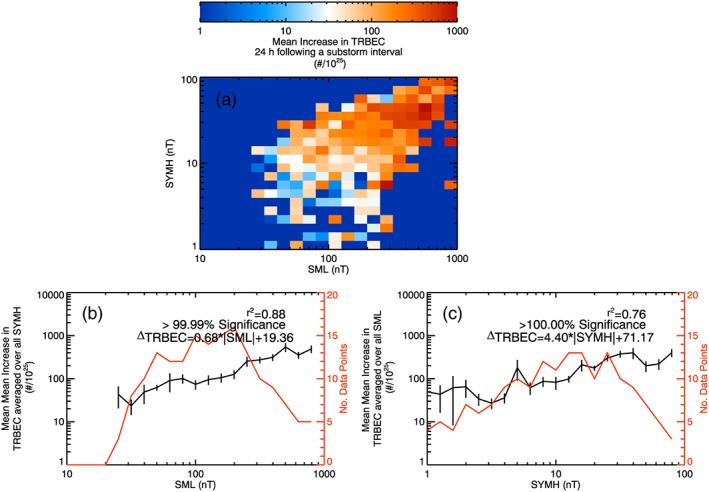
(a) Two‐dimensional histogram of the mean increase of TRBEC (divided by 10^25^) 24 h following substorm intervals against |SML| and |*SYM‐H*|. (b) The mean increase in TRBEC averaged over all |*SYM‐H*| against |SML|. (c) The mean increase in TRBEC averaged over all |SML|. The red traces in Figures 5b and 5c show the number of data points making up the mean values. The square of the Rank Order Correlation coefficient is given in Figures 5b and 5c, along with the significance of this correlation determined by the Student's *T* test.

**Figure 6:**
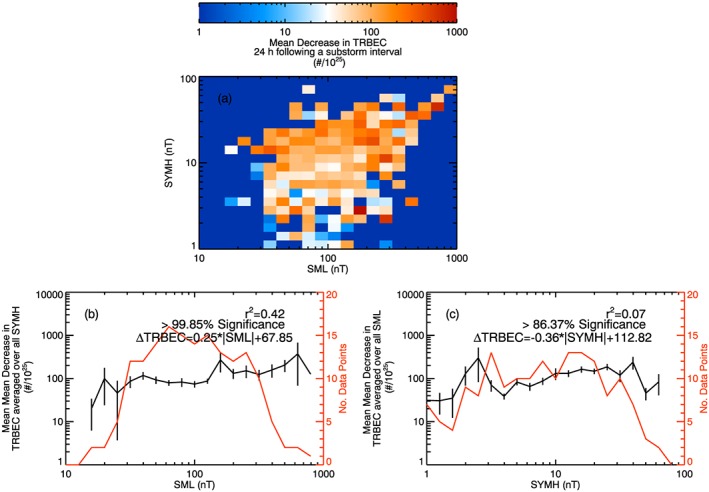
(a) Two‐dimensional histogram of the mean decrease of TRBEC (divided by 10^25^) 24 h following substorm intervals against |SML| and |*SYM‐H*|. (b) The mean increase in TRBEC averaged over all |*SYM‐H*| against |SML|. (c) The mean increase in TRBEC averaged over all |SML|. The red traces in Figures 6b and 6c show the number of data points making up the mean values. The square of the Rank Order Correlation coefficient is given in Figures 6b and 6c, along with the significance of this correlation determined by the Student's *T* test.

Given that substorm occurrence enhances the mean change of TRBEC, we examine whether a measure of substorm activity is linked with the change of TRBEC following substorms. Figure 5 shows (a) a 2‐D histogram of the mean increase in TRBEC against 3 h mean SML and 3 h mean *SYM‐H*, (b) the mean increase in TRBEC averaged over all SML plotted against 3 h mean *SYM‐H*, and (c) the mean increase in TRBEC averaged over all *SYM‐H* plotted against 3 h mean SML. The increases are calculated for a lag of 24 h from the measurements of *SYM‐H* and SML. The red traces in Figures 5b and 5c show the number of data points in each mean value. Figure 6 presents the decreases in TRBEC against SML and SYM following a substorm in a similar fashion.

Figure 5 shows that the increase in TRBEC is greater for higher SML and *SYM‐H*. Figures 5b and 5c show that, on average, the increase in the mean TRBEC averaged over all *SYM‐H* shows a 88% linear correlation with SML, whereas averaging over all SML gives a 76% correlation with *SYM‐H*, although both are significant beyond the 99.9% level from the Student's *T* test. Correlating the raw data gives correlations of ~30% using the Spearman's Rank Order Correlation for both SML and *SYM‐H*, as one would expect from Figure 1. This implies that, on average, the size of substorms is a better indicator of the subsequent change in the radiation belt content than the storm size, although fully deconvolving storm and substorm effects from these data may not be possible.

Figure 6 shows the same analysis for decreases in TRBEC following substorms and increases and decreases in TRBEC following quiet intervals. For these data, there is only a 42% correlation between the mean decreases in TRBEC following substorms and SML, which is much lower than the correlation shown above for increases in TRBEC following substorms. Similarly, there is only a correlation of 7% between decreases in TRBEC and *SYM‐H* following substorms. The statistical significance of the SML correlation was slightly below the 99.9% level, but there was no significant correlation between decreases in TRBEC and *SYM‐H*.

Changes in TRBEC showed no significant correlations with SML or *SYM‐H* following quiet intervals. For brevity, these data are presented in the supporting information for the interested reader.

In summary, our results show that the radiation belts are inherently lossy such that the median change in the radiation belt content of 1000–2000 MeV/G electrons is negative following both quiet and substorm intervals. Following a quiet interval, there is up to a 75% chance that the radiation belt content will decrease; however, following substorms, there is up to a 50% chance that they will increase. The difference in the proportion of increases and decreases following substorm or quiet intervals is significantly different up to 6 days after the fact, with this difference peaking after 0.5–2.5 days. The mean change in TRBEC following substorms shows a broad peak centered on 48 h after the event window, in keeping with the cross correlation between SML and TRBEC. This indicates that there can be a lag between the occurrence of magnetospheric activity and its effect on the radiation belts or that radiation belts have a memory of substorm and quiet activity. Furthermore, the distribution of increases in the radiation belt content is found to be enhanced following substorm intervals with respect to quiet intervals and the increases in the radiation belt content showed a stronger correlation with substorm activity levels than storm activity levels. Substorms are thought to be a key component of radiation belt energization, providing the injection of a seed population of keV electrons and enhancing wave power in the inner magnetosphere that then accelerates this seed population to MeV energies [*Baker et al*., 1998; *Horne and Thorne*, 1998; *Meredith et al*., 2001, 2002, 2003a; *Horne et al*., 2005a, 2005b; *Jaynes et al*., 2015]. Our results show that the occurrence of increases in the radiation belts is enhanced following substorm times compared to quiet times and that the increase in the radiation belt content is related to the level of substorm activity, although the extent to which losses are dependent on substorm activity levels is much weaker. As such there are nuances in substorm processes that must be taken into account if we wish to understand how substorms affect the radiation belts.

## 3 Discussion

By examining the radiation belt content following both quiet and substorm intervals, we have shown that the radiation belts are inherently lossy. Following quiet intervals, there is up to a 75% chance of there being a loss of particles from the radiation belts with average loss rates from 20 to 200 × 10^25^ el d^−1^. The effect of substorms is to reduce the likelihood of the radiation belt content decreasing, or alternatively the effect of substorms is to increase the likelihood of the radiation belt content increasing. The result of this can be seen in Figure 1, particularly on long time scales, with relatively short duration increases followed by an extended period of decreasing TRBEC. The average rate of decrease was higher following substorms than following quiet intervals for the first 24 h after the event, implying that substorm activity enhances losses as well as increases in the radiation belt content. This is consistent with increases in plasmaspheric hiss, whistler mode, and electromagnetic ion cyclotron (EMIC) wave activity which can enhance pitch angle scattering rates [e.g., *Tsurutani and Smith*, 1974; *Meredith et al*., 2004; *Usanova et al*., 2012]; however, the average losses showed no correlation with substorm activity levels. Due to the nature of the observations, we do not measure the enhancement and loss rates directly; instead, we examine the net change in the radiation belt content. Hence, we cannot separate out the increase and loss rates and our results should thus be taken as such.

Our results presented above clearly show that some substorm intervals result in increases in the radiation belt while others do not. One reason that a substorm may not lead to an increase in the radiation belts is that particles injected toward the inner magnetosphere during substorms do not get sufficiently close to the Earth to provide a seed population for the wave generation and acceleration processes. *Boakes et al*. [2009] showed that for a subset of 135 events in the *Frey et al*. [2004] list, only ~50% of substorms showed the signature of a clear injection of electrons into geosynchronous orbit and ~25% showed no injection signature at all. *Sergeev et al*. [2012] discussed that the injection of particles into the inner magnetosphere depends on the injected particle population having a similar entropy to the plasma in the inner magnetosphere in order to penetrate into the radiation belt region via the interchange instability. Thus, this does not necessarily mean that 50% of events do not have injections but may indicate that these injections do not penetrate into the inner magnetosphere, and hence, the injected particles are not accelerated up to high levels. *Jaynes et al*. [2015] discussed that if any of the necessary populations (particle or wave) were not present in the radiation belts, the radiation belts would not be enhanced. Thus, following 50% of the substorm intervals that we studied, either the substorms did not produce the necessary wave population to accelerate the seed population or the seed population was not injected into the radiation belts. Taking the *Boakes et al*. [2011] results to be a statistically representative subset of events, it is more likely that only 50% of substorms inject particles into the radiation belts.

Our results show a number of interesting features with regard to the time scales of acceleration and loss following substorms or quiet intervals. First, the radiation belts appear to be inherently lossy. After 33 h following a substorm interval, and for up to >200 h following a quiet interval, there are statistically significantly more decreases in the radiation belt that increases. This can, in fact, be seen in Figure 1a, with large‐scale increases in the radiation belts being sharp and short lived, while the decreases have a much longer period, giving a sawtooth‐like profile to the radiation belt content. Second, our analysis shows that on average, changes in the radiation belt content are lagged by ~1–3 days following magnetospheric activity or a lack thereof. Examining the occurrence of increases or decreases in TRBEC shows that the effect of substorms or quiet times is most significant at ~1 day following the event, whereas the mean changes in TRBEC are greatest after 48 h, as is the cross correlation between SML and TRBEC. Third, our analysis shows that the radiation belts have a memory of the substorm or quiet interval. The effect of substorms is to increase the likelihood of the radiation belts increasing 6 days after their occurrence. This implies that the acceleration mechanism is enhanced by the substorm activity but not limited to it. Conversely, the effect of quiet intervals is to increase the likelihood of the radiation belts decreasing for more than 6 days after the fact. This implies that a relatively short period with no substorm activity can suppress acceleration within the radiation belts for a far longer period afterward. In the context of wave‐particle interactions causing enhancements and losses in the radiation belts, it is interesting to ask what the lifetime of the seed population and whistler mode chorus and other waves are following substorm interval and quiet intervals. To date, we are not aware of any study that addresses this question.

Previous studies have reported that changes in electron flux at geosynchronous orbit at storm commencement are correlated with the size of the storm. *Moon et al*. [2004] examined the ratio of the electron fluxes before a storm and following the storm commencement for 50–400 keV electrons during 22 storm events and found that these correlated with the minimum storm time *Dst* index, with correlation coefficients (*r*) of 0.64–0.84 corresponding to 40–70% correlation (*r* ^2^). It is therefore unsurprising that the change in the higher‐energy population is also somewhat correlated with *SYM‐H* (a comparable measure to *Dst*), as shown in this study. However, the complex interplay between particle injection, wave generation, and wave‐particle interactions that results in different losses and gains in the high‐energy electron population means that the correlation between the changes in higher‐energy particle fluxes and *SYM‐H* does not necessarily reflect the correlations seen with lower energy particles. Furthermore, we consider the correlation between the increases in the radiation belt content and SML or *SYM‐H* for a far greater number of events.

The loss and acceleration mechanisms in the radiation belts are a complex interplay of different wave‐particle interactions as well as plasma transport. Our results show that losses and acceleration occur after both quiet and substorm intervals, although with a greater proportion of loss periods following quiet intervals and a greater proportion of acceleration intervals following substorms. From modeling, the time scale for the radiation belt electron flux to increase by an order of magnitude has been put at ~24 h [e.g., *Horne et al*., 2005a], whereas our results show that the mean change in TRBEC following substorms is positive and statistically significantly different to that following quiet intervals up to 96 h after the fact. Within that time there are likely to be both substorm and quiet intervals. As such, increases or decreases in the radiation belt content may also depend on activity prior to or following the reference interval. Take, for example, a quiet interval following a period of substorm activity: if the loss rate increases, it may not overcome the preexisting acceleration rate; thus, the overall result is an increase, albeit at a lower acceleration rate. However, if we similarly consider a substorm interval following a quiet interval, we would expect that the acceleration of particles would reduce any rate of decrease. This is not seen in the data. Rather than the distribution of changes in TRBEC being shifted toward increases in TRBEC following substorm intervals, the distribution of radiation belt losses is approximately the same following substorm and quiet intervals at the lags examined, with substorms providing an additional population of increases. As such, in order to predict changes in the radiation belts following substorms, one must be able to determine what controls whether or not the substorm provides enhancements in radiation belt.

*Meredith et al*. [2003a] suggested that a prolonged period of substorm activity may be needed to energize the radiation belts. Using data from the extended solar activity minimum at the end of solar cycle 23, *Rodger et al*. [2015] showed that recurrent substorms from the *Newell and Gjerloev* [2011] list (separated by less than 82 min) are more efficient that isolated substorms (separated by more than 3 h) in enhancing the radiation belts, although both recurrent and isolated substorms showed increase in the radiation belt electron flux. Within this study, we have considered substorm expansion and recovery phase occurrences within 3 h windows; thus, we do not separate isolated and recurrent substorms; however, we do show that the largest changes in radiation belt content occurred during periods of large *SYM‐H*, suggestive of storm times in which we would expect to see periods of recurrent substorms.

It is generally thought that whistler mode chorus waves are the dominant process that accelerates the seed population up to MeV energies [e.g., *Horne et al*., 2005a, 2005b; *Li et al*., 2007; *Thorne et al*., 2013; *Reeves et al*., 2013; *Jaynes et al*., 2015]. The amplitude of these waves relates to their ability to accelerate particles, and this has been shown to increase with *AE* and thus substorm activity [*Meredith et al*., 2001, 2003b]. However, whistler mode chorus waves are also implicated in pitch angle scattering that can move particles into the loss cone [*Horne and Thorne*, 2003]. Similarly, EMIC waves can efficiently scatter particles into the loss cone [*Meredith et al*., 2003c; *Bortnik et al*., 2006; *Hendry et al*., 2014; *Usanova et al*., 2014; *Rodger et al*., 2015]. A modeling study by *Li et al*. [2007] suggested that during storms the whistler mode chorus waves should give a net acceleration of particles, while EMIC waves were the dominant loss mechanism. However, both *Horne et al*. [2005a] and *Li et al*. [2007] used average wave power distributions observed during substorms determined by a single spacecraft (based on *Meredith et al*. [2001, 2003b]). These spatiotemporal averages can hide important information and are not necessarily representative of any individual event. Given that our results show that increases and decreases in the radiation belt content occur following substorms, the important question is how do the wave populations vary for increases and decreases in radiation belt content and how does this relate to substorm activity? Our results show radiation belt increases and decreases for all levels of substorm activity. However, while radiation belt increases are reasonably well correlated with SML, radiation belt decreases are less dependent on substorm size. This is somewhat counterintuitive if one expects the loss mechanisms to depend on wave amplitudes which, on average, increase with substorm size.

A number of studies have examined how the acceleration of radiation belt particles may be related to upstream solar wind conditions, thus giving a way in which to predict future variations in the radiation belt [e.g., *Baker et al*., 1979; *Reeves et al*., 2011; *McPherron et al*., 2009; *Li et al*., 2015; *Kim et al*., 2015]. The solar wind can directly influence the radiation belts through the generation of ULF waves and modifying the particle population to generate VLF waves [e.g., *Elkington*, 2006; *Shprits et al*., 2008; *Ebihara and Miyoshi*, 2011; *Miyoshi et al*., 2013] or can indirectly influence the radiation belts by enhancing the energy of the plasma sheet population prior to substorms that are then subsequently injected [*Forsyth et al*., 2014; *Sergeev et al*., 2015]. In this study, we have not considered the impact of the solar wind on the radiation belts but rather statistically examined whether substorm activity alone shows any correspondence to changes in the radiation belt. It is worth noting that many of the studies that have examined the solar wind impact on the radiation belts show that the solar wind conditions that lead to radiation belt acceleration include a prolonged southward interplanetary magnetic field, elevated solar wind speed, but low solar wind density (thus giving a low dynamic pressure). These solar wind conditions are similar to those that are conducive to causing storms and substorms [e.g., *Gonzalez and Tsurutani*, 1987; *Morley and Freeman*, 2007], and as such the link between solar wind conditions and increases in the radiation belts may be both direct and indirect.

It should not be a surprise that the response of the radiation belts shows a multifaceted response to substorms, given that the radiation belts can be energized, depleted, or unchanged during storms [*Reeves et al*., 2003]. In fact, our results show some similarity with those of *Reeves et al*. [2003], in that only 50% of substorms resulted in an increase in the radiation belt content, in keeping with their result that only 50% of storms show an increase in the radiation belt electron fluxes. However, our results cover a large range of *SYM‐H* values showing that both storm time and non–storm time substorms show increases and decreases in the radiation belts.

## 4 Conclusions

We have statistically compared changes in the total radiation belt electron content from the Van Allen Probes over time with substorm activity determined by the SOPHIE algorithm. Substorm activity was broken down into 3 h windows in which substorm expansion or recovery phases began (substorm intervals) or there were no expansion and recovery phase onsets (quiet intervals). Changes in the radiation belt content were calculated as a net change over increasing time intervals. Our results show the following:

1. There is a 50% chance of an increase or decrease in the radiation belt content up to 33 h following a substorm interval.
2. There is up to a 75% chance of a decrease in radiation belt content following quiet intervals.
3. The radiation belts have an apparent memory of substorms and quiet intervals, extending out to 6 days after the event.
4. Substorms and quiet intervals are good predictors of increases and decreases in radiation belt with this skill and accuracy of this prediction maximizing at a lag of between 0.5 and 2.5 days.
5. The increases in radiation belt content 24 h following substorm intervals are correlated with both SML and *SYM‐H*, along the correlation with SML is stronger.

Furthermore, we have provided the median increases in TRBEC 24 h following a substorm for given ranges of SML and *SYM‐H*.

These results raise important questions for the existing framework for substorms increasing the radiation belt contents, namely, what prevents half of substorm intervals from increasing the radiation belt content and what controls the radiation belt loss rate? These are fundamental questions that must be answered in order to develop accurate modeling of the radiation belts with respect to substorm activity.

## Supporting information

Supporting Information S1

Figure S1

Figure S2
