# Supplementary material for: What effect do substorms have on the content of the radiation belts?
Source: J Geophys Res Space Phys. 2016 Jul 9;121(7):6292–306. doi: 10.1002/2016JA022620 (PMC5014235; doi:10.1002/2016JA022620)
Supplement: Supplementary file 1 — Supporting Information S1 [file JGRA-121-6292-s001.docx]

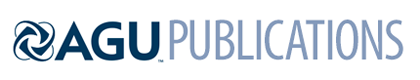


*Journal of Geophysical Research – Space Physics*

Supporting Information for

**What effect do substorms have on the content of the radiation belts?**

C. Forsyth[1], I. J. Rae[1], K. R. Murphy[2], M. P. Freeman[3], C. L. Huang[4], H. E. Spence[4], A. J. Boyd[4], J. C. Coxon[5], C. M. Jackman[5], N. M. E. Kalmoni[1], C. E. J. Watt[6]

[1] UCL Mullard Space Science Laboratory, Dorking, UK
[2] NASA Goddard Space Flight Center, Greenbelt, Maryland, USA
[3] British Antarctic Survey, Cambridge, UK
[4] University of New Hampshire, Durham, New Hampshire, USA
[5] University of Southampton, Southampton, UK
[6] University of Reading, Reading, UK

**Contents of this file**

Text S1

Table S2

Figures S3 to S4

**Introduction**

In the paper “What effect do substorms have on the content of the radiation belts?” we examine how the total radiation belt electron content (TRBEC) of 1000-2000 MeV/G electrons varies with respect to substorm occurrence and the correlation between SML, SYMH and TRBEC. In the paper, we use Contingency Tables to examine whether substorms and quiet intervals have a statistically significant effect on the occurrence of increases or decreases in TRBEC. We also assess how good substorms and quiet intervals are at predicting increases and decreases in TRBEC using the Heidke Skill Score [Heidke, 1926] and the accuracy. The methodology for this analysis is outline in this Supplementary Information. Furthermore, we discuss in the paper that increases and decreases in TRBEC appear to be correlated with SML and SYMH following substorms but increases and decreases following quiet intervals so no apparent link with SML or SYMH. In this Supplementary Information, we show 2D histograms of the increases and decreases in TRBEC following quiet intervals (as defined in the paper). These figures are in the same format as Figure 5 and 6 in the paper.

Text S1: The use of Contingency Tables

Contingency Tables are a standard statistical tool that enables the frequency distribution of a multi-variate data set and are commonly used in many branches of statistical analysis. From these tables, it is possible to determine whether there is a statistically significant link between the variables tested. Here, we provide a brief overview of there use in our paper. This is not a substitute for the greater details given in text books on this subject, to which we direct the interested reader.

At any time, we can determine whether the total radiation belt content (calculated from Van Allen Probe data) is increasing or decreasing. Similarly, using the SOPHIE substorm detection algorithm (Forsyth et al., 2015), we can determine whether there was or was not substorm activity at that time. Thus we can determine the frequency distribution of increases at substorm times, increases at non-substorm times and similarly decreases at substorm times and decreases at no substorm times. In the paper, we perform this analysis looking at increases and decreases in TRBEC at different time lags away from the substorm or quiet intervals, thus can determine the time dependence of increases and decreases in TRBEC following substorm and quiet intervals.

One of the benefits of this analysis is that based on the various frequency distributions, we can calculated the expected frequency distribution for the null hypothesis that increases or decreases in TRBEC are not dependent on substorms and quiet intervals. For each cell in the table, the expected frequency is calculated as

$$Expected Frequency=\frac{Column Total \times Row Total}{Table Total}$$

From the observed and expected frequencies, we can calculate the χ^2^ statistic to assess the statistical significance in any difference in the occurrence frequencies.

$$\chi^{2}=\sum\frac{{(Observed-Expected)}^{2}}{Expected}$$

An example Contingency Table is shown in Table 1 in the paper and reproduced below.

Table S2*: Contingency table of TRBEC increases and decreases 24 hrs following substorm or quiet intervals.*

|  | Observed values | | Proportion of substorm/quiet observations | | Expected values for null hypothesis (no relation between increases or decreases with substorms or quiet intervals) | |
| --- | --- | --- | --- | --- | --- | --- |
|  | TRBEC Increase | TRBEC decrease | TRBEC Increase | TRBEC decrease | TRBEC Increase | TRBEC decrease |
| Substorm Interval | 851 | 838 | 0.504 | 0.496 | 714 | 975 |
| Quiet Interval | 303 | 738 | 0.291 | 0.709 | 440 | 601 |

Calculating Accuracy and Heidke Skill Score

If we take substorms to be predictors of increases in TRBEC and quiet intervals to be predictors of decreases in TRBEC, then from the above contingency table we can calculate the accuracy of substorms or quiet intervals forecasting the changes in TRBEC. We are also able to calculate the Heidke Skill Score [Heidke, 1926], which the extent to which the forecast is better than random chance. These measures are commonly used in assessing weather forecasting and have recently been used to compare different techniques for identifying the magnetotail lobes [Coxon et al., 2016].

The accuracy of substorms or quiet times predicting increases or decreases in TRBEC is simply calculated as the number of correct predictions (top left and bottom right cells of the table) divided by the total number of observations

$$\boldsymbol{Accuracy=}\frac{\left( \boldsymbol{substorms with increases+quiet with decreases} \right)}{\boldsymbol{total number of events}}$$

The Heidke Skill Score (HSS) gives an indication of the skill of substorms or quiet times in predicting increases or decreases in TRBEC and the extent to which the results are an improvement on random chance. The HSS has a range of -∞ < HSS < 1, with an HSS of 0 showing no skill greater than random chance and a HSS of 1 showing perfect skill. HSS is calculated as

$$\boldsymbol{HSS=}\frac{\boldsymbol{2}\left( \boldsymbol{s}\boldsymbol{s}_{\boldsymbol{inc}}\boldsymbol{\times}\boldsymbol{q}_{\boldsymbol{dec}}\boldsymbol{-s}\boldsymbol{s}_{\boldsymbol{dec}}\boldsymbol{\times}\boldsymbol{q}_{\boldsymbol{inc}} \right)}{\left( \boldsymbol{s}\boldsymbol{s}_{\boldsymbol{inc}}\boldsymbol{+s}\boldsymbol{s}_{\boldsymbol{dec}} \right)\left( \boldsymbol{s}\boldsymbol{s}_{\boldsymbol{dec}}\boldsymbol{+}\boldsymbol{q}_{\boldsymbol{dec}} \right)\boldsymbol{+}\left( \boldsymbol{s}\boldsymbol{s}_{\boldsymbol{inc}}\boldsymbol{+}\boldsymbol{q}_{\boldsymbol{inc}} \right)\left( \boldsymbol{q}_{\boldsymbol{inc}}\boldsymbol{+}\boldsymbol{q}_{\boldsymbol{dec}} \right)}$$

Where *ss* indicates a substorm frequency, *q* indicates a quiet interval frequency and the subscripts *inc* and *dec* indicate increase or decrease frequencies.


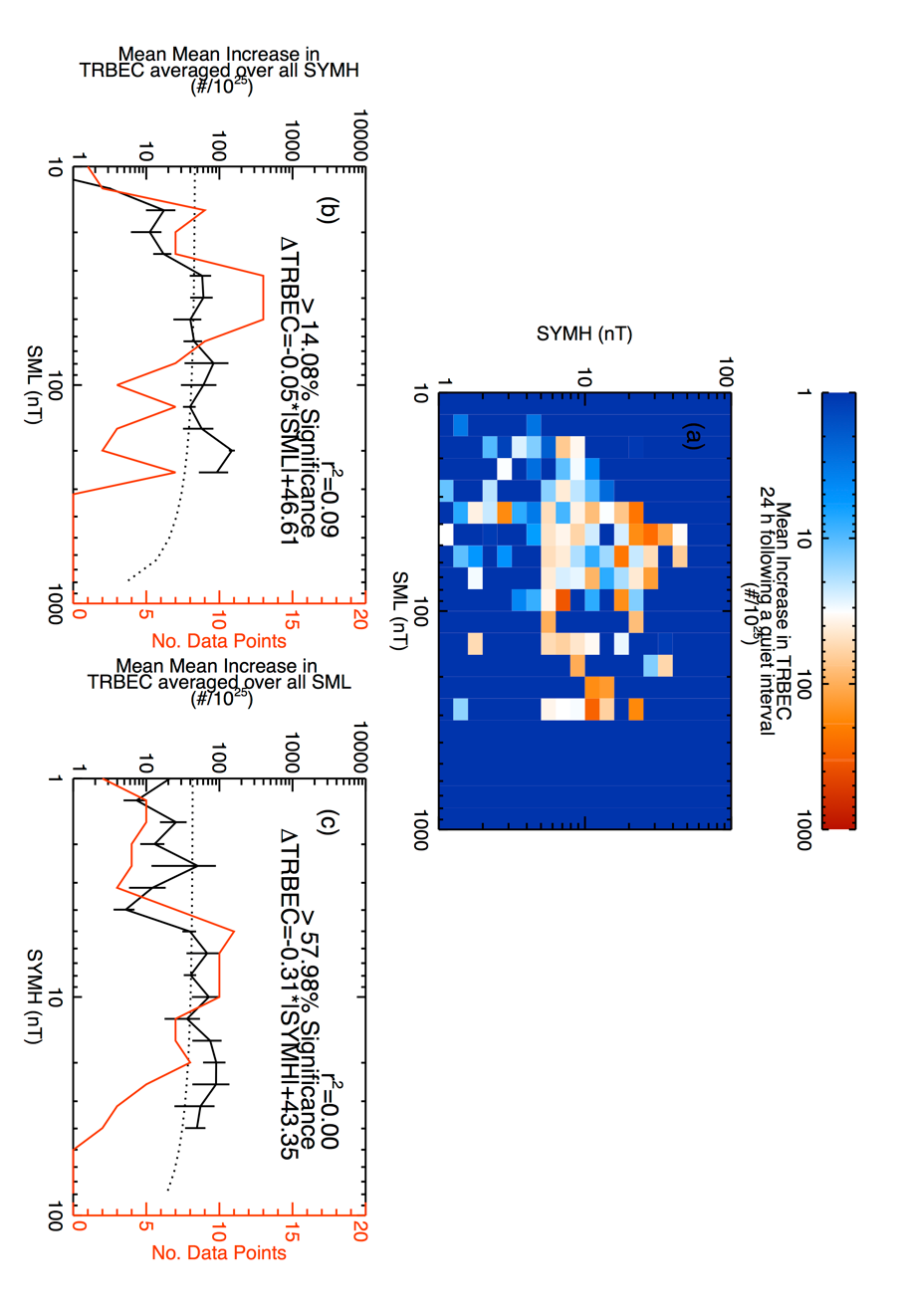


Figure S3: (a) 2D histogram of the mean increase of TRBEC (divided by 10^25^) 24 hrs following quiet intervals against |SML| and |SYMH|. (b) The mean mean increase in TRBEC averaged over all |SYMH| against |SML|. (c) The mean mean increase in TRBEC averaged over all |SML|. The red traces in Panels (b) and (c) show the number of data points making up the mean values. The square of the Rank Order Correlation coefficient is given in (b) and (c), along with the significance of this correlation determined by the Student’s T-Test. The correlations of TRBEC against both |SML| and |SYMH| are weak and are not statistically significant.


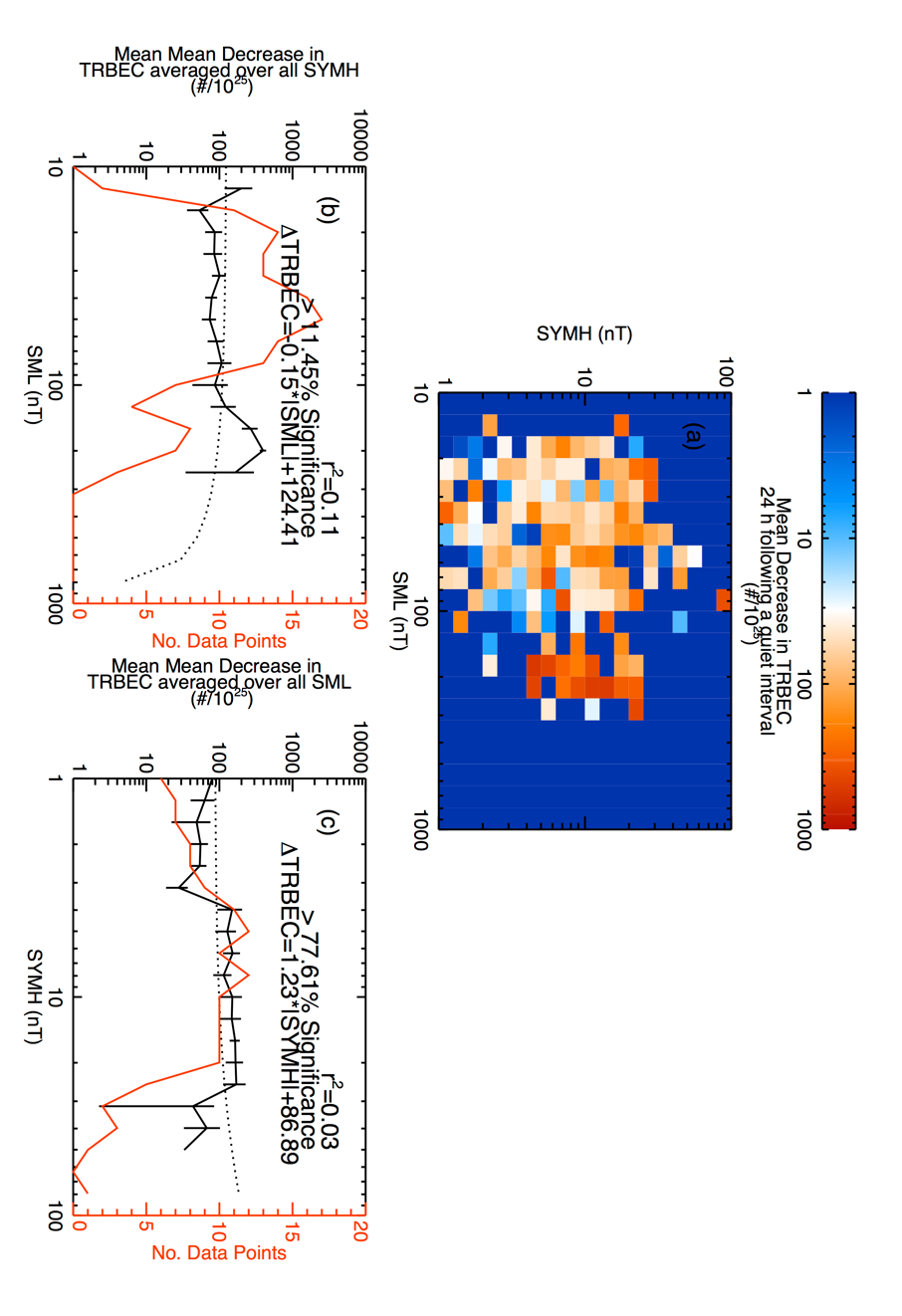


Figure S4: (a) 2D histogram of the mean decrease of TRBEC (divided by 10^25^) 24 hrs following quiet intervals against |SML| and |SYMH|. (b) The mean mean decrease in TRBEC averaged over all |SYMH| against |SML|. (c) The mean mean decrease in TRBEC averaged over all |SML|. The red traces in Panels (b) and (c) show the number of data points making up the mean values. The square of the Rank Order Correlation coefficient is given in (b) and (c), along with the significance of this correlation determined by the Student’s T-Test. The correlations of TRBEC against both |SML| and |SYMH| are weak and are not statistically significant.
